# Phylogenetic Analysis and Molecular Typing of Trichothecene-Producing Fusarium Fungi from Russian Collections

**Published:** 2018

**Authors:** A. A. Stakheev, L. V. Samokhvalova, O. D. Mikityuk, S. K. Zavriev

**Affiliations:** M.M. Shemyakin and Yu.A. Ovchinnikov Institute of Bioorganic chemistry of the Russian Academy of Sciences, Miklukho-Maklaya Str. 16\10, Moscow, 117997, Russia; All-Russian Research Institute of Phytopathology, Institut Str. 5, B. Vyazyomy, Moscow region, 143050 , Russia

**Keywords:** Fusarium, trichothecene mycotoxins, DNA markers, phylogenetic analysis, identification, chemotype

## Abstract

We performed a three-locus phylogenetic analysis of *Fusarium *strains presumably capable of trichothecene production, which were deposited in the Russian national collections. The intra- and interspecific polymorphism of partial sequences of the translation elongation factor 1 alpha (*TEF1α*) gene and two genes from the trichothecene cluster *TRI5 *and *TRI14 *was studied. A study of 60 strains of different origins using DNA markers confirmed, and in the case for several strains, clarified their taxonomic characteristics. As a result, a strain of *F. commune *(F-900) was identified in Russia for the first time. Furthermore, the strain F-846 proved to be phylogenetically distinct from any of the known *Fusarium *species. *F. equiseti *strains from Northwest Russia were found to belong to the North European group (I), whereas a strain from the North Caucasus – to the South European one (II). Partial *TRI14 *sequences from 9 out of 12 species were determined for the first time. Their comparative analysis demonstrated a relatively high level of intraspecific variability in *F. graminearum *and *F. sporotrichioides*, but no correlation between the sequence polymorphism and the geographic origin of the strains or their chemotype was found. Specific chemotypes of trichothecene B producers were characterized using two primer sets. The chemotyping results were verified by HPLC.

## INTRODUCTION

Fungi of the genus *Fusarium *from the class *Ascomycetes *occupy various ecological niches and occur in various climatic zones. In Russia, *Fusarium *species are ubiquitous in all regions where agricultural crops, primarily cereals, are grown. This fungus causes significant damage to the agricultural and food industries, resulting in several hundred million dollar losses annually. In addition, the mycotoxins produced by members of the genus *Fusarium *pose a threat to human and animal health and also act as pathogenicity factors for plants [1].

Trichothecene mycotoxins (TrMTs) are the most extensive group of toxic metabolites produced by *Fusarium *fungi. Trichothecene mycotoxins are produced not only by members of the genus *Fusarium, *but also by those from the genera *Myrothecium*, *Trichoderma*, *Cephalosporium*, *Verticimonosporium*, and *Stachybotrys* [2]. To date, about 200 trichothecene toxins have been identified [3-6]. TrMTs are sesquiterpene compounds consisting of three rings with an epoxide ring at the C-12–C-13 atoms and a double bond at the C-9–C-10 atoms: so, the group is called 12,13-epoxy-trichotec-9-ens. Depending on the side group structure, trichothecene toxins are divided into four types (A–D), with only types A and B produced by *Fusarium *fungi. More toxic members of type A trichothecenes include diacetoxyscirpenol (DAS), as well as toxins T-2 and HT-2, and the main producers of these toxins are *F. sporotrichioides *and *F. langsethiae*. In 2015–2016, a new group of type A trichothecene toxins, named NX, was described [7, 8]. Interestingly, these compounds were identified in cultures of *F. graminearum*, a traditional producer of B-type TrMTs. Type B is represented by compounds such as nivalenol (NIV), deoxynivalenol (DON), and their acetylated derivatives (3- and 15-ADON and 4,15-ANIV) that are produced by *F. graminearum*, *F. culmorum*, *F. cerealis*, and a group of species known as the *F. graminearum *species complex (FGSC) [9, 10]. In addition, *F. poae*, *F. venenatum*, and *F. equiseti *are capable of producing both type A and type B toxins [11]. The type of toxins produced by a particular strain is determined by the structure and functions of the genes present in the trichothecene cluster [12, 13]. In most trichothecene-producing *Fusarium *species, the main cluster comprises 12 genes that encode both of the enzymes responsible for different biosynthesis stages and regulatory factors, some of which control the expression of a large number of the genes associated with various aspects of the fungal metabolism and life activity [14]. Trichothecene toxins inhibit protein synthesis in eukaryotes [15], and trichothecenes such as DON are important aggressiveness factors promoting fungal spread within host plant tissues. Artificial inoculation of cereal ears with mutant *F. graminearum *strains with abolished DON synthesis has been shown to infect a smaller number of kernels compared to inoculation with wild-type strains [16].

The danger posed by *Fusarium *fungi and their mycotoxins necessitates the development of methods for a quick and reliable species-specific identification of the strains which will enable a determination of the spectrum of the compounds contained in a culture or batch of grain. At present, DNA polymorphism analysis methods play an important role in taxonomic studies of the genus *Fusarium *and in the identification of its members. Application of the molecular genetics approach has enabled a clarification of the standards and boundaries of species, as well as the characterization of several new taxa. In particular, a multilocus phylogenetic analysis using sequence-characterized amplified region (SCAR) markers [17] based on 13 housekeeping genes enabled the identification of 9 new species within the FGSC [10], which had been previously considered as a single-species *F. graminearum*. A little later, species *F. vorosii *and *F. gerlachii *were also included in this complex [18]. In total, 16 phylogenetic species can be distinguished in the FGSC [19]. The phylogenetic approach was used to confirm the status of *F. pseudograminearum *and *F. culmorum *as separate species [20, 21]. In Russia, an analysis of polymorphic DNA markers enabled the identification of strain groups that were subsequently described as two new species: *F. ussurianum*, morphologically and phenotypically similar to *F. graminearum* [22], and *F. sibiricum*, closely related to *F. sporotrichioides* [23]. A number of recent phylogenetic studies have determined an intricate structure of the *F. equiseti*–*F. incarnatum *species complex (FIESC) and identified several new species within the complex [24]. In addition, investigation of inter- and intraspecific DNA polymorphisms has made possible the development of several highly specific diagnostic and identification systems for the main *Fusarium *pathogens, which are primarily based on PCR and its modifications [25-29]. The use of modern molecular biological and bioinformatic methods, including whole genome sequencing [30, 31], has significantly accelerated the investigation of the genetic diversity of the genus *Fusarium *and the functional characterization of genomic elements, but the search for effective methods of molecular typing and informative DNA barcodes still remains topical [32, 33].

The genus *Fusarium *is different from most other taxa of the kingdom Fungi. The “gold standard” of molecular fungal taxonomy is the ribosomal DNA internal transcribed spacer (ITS) region [34]. However, these markers in the genome of members of the genus *Fusarium *are represented by two nonorthologous copies and do not possess a sufficient level of interspecific polymorphism [35]. Today, the *TEF1α *gene is most often used as a marker in phylogenetic and taxonomic studies [36, 37]. It seems promising to use the genes involved in mycotoxin biosynthesis as phylogenetic markers. For example, the trichodiene synthase gene (*TRI5*) has been used to develop species-specific primer systems [38] and to study intraspecific polymorphism in members of the *F. equiseti *species complex [39, 40]. However, the phylogenetic characteristics of the *TRI5 *gene have not been compared with those of “classical” markers, such as *TEF1α*. Among other genes that comprise the trichothecene cluster and are used in phylogenetic studies, it is necessary to emphasize the role of *TRI1 *encoding cytochrome P450 monooxygenase and *TRI12 *encoding a trichothecene efflux pump [41]. A phylogenetic analysis of the *TRI1 *gene helped to identify a group of *F. graminearum *strains capable of producing the NX-2 toxin [7]. *TRI12 *polymorphism was used to design primers for the detection of type B TrMT-producing strains based on their chemotype (3/15-ADON, NIV) [42]. The trichothecene cluster also includes a number of genes that have not been characterized either structurally or functionally; e.g., *TRI9 *and *TRI14 *[43].

*Fusarium *strains from Russian national collections, which are potentially capable of producing TrMTs and represent different climatic and geographic regions of Russia, have not been characterized by molecular genetic methods. Therefore, the main objectives of this study were as follows: (1) a SCAR marker-based analysis of the accuracy of the taxonomic identification of the trichothecene-producing *Fusarium *strains deposited in Russian national collections; (2) an investigation of the molecular genetic diversity of strains of different geographical origin, isolated in different years and from different sources; (3) determination of the interand intraspecific polymorphism of the *TRI14 *gene, one of the least studied genes of the trichothecene cluster; and (4) determination of chemotypes of type B TrMT-producing strains using specific PCR primers, with verification of the data by HPLC.

## EXPERIMENTAL

**Fungal strains**

We analyzed 60 strains of 12 *Fusarium *genus species deposited in Russian national collections and presumably possessing ability for TrMT biosynthesis. The choice of the strains was based on maximum coverage of various natural and geographical zones of Russia. In addition, the study included strains from a number of neighboring countries, as well as from Moldova and Germany. We also studied the *F. graminearum* strain F-892 (VKPM) deposited in the StrainInfo database (http://www.straininfo.net; ATCC 36015). The list of strains with indication of their geographical origin, host plant species, year of isolation, and the particular collection are given in *Table 1*. In addition, the morphological features of several strains the initial identification of which had not been confirmed by molecular methods were determined using a MIKMED 6 laboratory microscope (Lomo, Russia). For the microscopic analysis, the fungal strains were grown on carnation leaf agar (CLA) and synthetic nutrient deficient agar (SNA; Nirenberg) for 10–14 days.

**DNA isolation**

Prior to DNA isolation, monospore cultures of fungi were grown on potato sucrose agar (PSA) at room temperature for 10 days, until abundant mycelium was obtained. DNA was isolated from monospore fungal cultures by a method based on the use of cetyltrimethylammonium bromide as a detergent, with allowance for the modifications described earlier [28]. The concentration and purity of DNA samples were evaluated using a NanoVue spectrophotometer (GE HealthCare, USA).

**Design of universal primers, PCR, and sequencing**

To amplify partial sequences of the *TEF1α*, *TRI5*, and *TRI14 *genes, we constructed the following primer pairs: TEF50F (5’-CGACTCTGGCAAGTCGACCAC-3’) and TEF590R (5’-CTCGGCTTTGAGCTTGTCAAG-3’); TRI5F (5’-ACACTGGTTCTGGACGACAGCA-3’) and TRI5R (5’-CCATCCAGTTCTCCATCTGAG-3’); TRI14F (5’-GAAGCTGCCTCGACATGGCTC-3’) and TRI14R (5’-AATAATATTATGGGGAACAATCAT-3’).

The primers were designed using the ClustalW algorithm [44]. The physicochemical properties of the primers were evaluated using the Oligo 6.71 software.

PCR was performed using the following amplification programs.

Primers TEF50F–590R: 93°C, 90 s; 93°C, 20 s; 64°C, 5 s; 67°C, 5 s (5 cycles); 93°C, 1 s; 64°C, 5 s; 67°C, 5 s (40 cycles).

Primers TRI5F–R and TRI14F–R: 93°C, 90 s; 93°C, 10 s; 55°C, 15 s; 72°C, 10 s (40 cycles).

PCR and electrophoretic analysis were performed according to [27, 28].

PCR products were cloned using an InstA Clone PCR cloning kit (Fermentas, Lithuania) according to the manufacturer’s protocol. DNA was sequenced at the Evrogen JSC using an ABI PRISM BigDye Terminator v.3.1 kit, followed by an analysis of the reaction products on an ABI PRISM 3730 automatic sequencer (Applied Biosystems).

The nucleotide sequences characterized in the present work are deposited in the NCBI GenBank under the accession numbers MG989711-989751 (*TEF1α*), MH001611-001651 (*TRI5*), and MH001652-001692 (*TRI14*).

**Phylogenetic analysis**

DNA markers with a characterized nucleotide sequence were compared with sequences deposited in the NCBI GenBank and Fusarium MLST databases (http://www.westerdijkinstitute.nl/fusarium/) using the BLAST algorithm. Phylogenetic trees were constructed with the maximum likelihood (ML) method and GTR+G (General Time Reversible) nucleotide substitution model [45] using the MEGA5.1 software [46]. In addition to the studied strains, several sequences of the appropriate genes of typical strains from international collections deposited in databases were used in the phylogenetic analysis. The reliability of phylogenetic tree topologies was confirmed by bootstrap analysis from 1,000 replicates. Insertions and deletions were omitted from the analysis. The number of variable, parsimony informative nucleotides and haplotypes for each marker was calculated with the DnaSP v6 software [47] using a sample of 41 strains.

**Molecular typing of type B TrMT producers**

To determine the chemotypes of the type B TrMT producers, we analyzed *F. graminearum*, *F. culmorum*, *F. cerealis*, and *F. ussurianum *strains. Chemotype-specific PCR was performed using three primer sets: two sets of primers for the polymorphic regions of the *TRI12 *gene [42] (the set is denoted as 12-1), [48 (12-2)], and a pair of primers for the amplification of *TRI13 *gene fragments with different lengths, depending on the chemotype [49 (13-1)]. The structure of primers and their melting temperatures are presented in *Table 2*.

**Table 1:** Fusarium fungal strains used in the study.

No.	Accession number	Species	Origin	Source	Year of isolation
1	M-99-43*	F. culmorum	Moscow Region	Wheat	1999
2	09-1/7*	F. culmorum	Moscow Region	Wheat	2009
3	M-99-9*	F. culmorum	Moscow Region	Wheat	1999
4	M-10-1*	F. culmorum	Moscow Region	Wheat	2010
5	BR-03-19*	F. culmorum	Bryansk Region	Wheat	2003
6	BR-0453*	F. culmorum	Bryansk Region	Wheat	2004
7	OM-0233*	F. culmorum	Omsk Region	Wheat	2002
8	OR-02-37*	F. culmorum	Orel Region	Wheat	2002
9	CM-9864*	F. culmorum	Smolensk region	Wheat	1998
10	KP-1136-66*	F. culmorum	Kirov region	Wheat	1995
11	KP-1599-25/3*	F. culmorum	Kirov Region	Wheat	1996
12	KS-1384-1*	F. culmorum	Kirov Region	Wheat	2007
13	M-05-111*	F. culmorum	Moscow Region	Wheat	2005
14	KC-1716-8*	F. culmorum	Kirov Region	Wheat	1997
15	58801**	F. culmorum	Moscow Region	Wheat	2004
16	Kz-27*	F. culmorum	Kostanay Region, Kazakhstan	Unknown	2014
17	74007**	F. culmorum	Arkhangelsk Region	Potato	Unknown
18	58030**	F. culmorum	Rostov Region	Cirsium	2004
19	70505**	F. culmorum	Belarus	Wheat	2003
20	50106**	F. culmorum	Leningrad Region	Cirsium	2006
21	64722**	F. cerealis	Khabarovsk Region	Wheat	2006
22	39295**	F. cerealis	Heilongjiang province, China	Wheat	2003
23	37032**	F. cerealis	Heilongjiang Province, China	Wheat	2003
24	39142**	F. cerealis	Heilongjiang Province, China	Wheat	2003
25	37031**	F. cerealis	Heilongjiang Province, China	Wheat	2003
26	41727**	F. cerealis	North Ossetia	Cirsium	2004
27	G.8-8**	F. graminearum	Germany	Wheat	1998
28	41806**	F. graminearum	North Ossetia	Wheat	2004
29	48702**	F. graminearum	Tula Region	Wheat	Unknown
30	58033**	F. graminearum	Leningrad Region	Wheat	2002
31	70725**	F. graminearum	Orel Region	Wheat	2006
32	58212**	F. ussurianum^1^	Primorsky Krai	Wheat	Unknown
33	29813**	F. ussurianum	Jewish Autonomous Oblast	Wheat	2002
34	MM-7*	F. sporotrichioides	Moscow Region	Wheat	2010
35	KG-9744*	F. sporotrichioides	Kirov Region	Wheat	Unknown
36	78105**	F. sporotrichioides	Orel Region	Wheat	2006
37	SK-1506*	F. sporotrichioides	North Ossetia	Wheat	2010
38	64706**	F. sporotrichioides	Primorsky Krai	Barley	2006
39	33100**	F. sporotrichioides	Primorsky Krai	Wheat	2003
40	74006**	F. sporotrichioides	Leningrad Region	Barley	2006
41	11007**	F. sibiricum	Krasnoyarsk Region	Barley	2000
42	11014**	F. sibiricum	Amur Region	Oat	2001
43	55201**	F. langsethiae	Kaliningrad Region	Oat	2005
44	82901**	F. langsethiae	Orel Region	Oat	2003
45	47401**	F. poae	Moscow Region	Wheat	2004
46	61701**	F. poae	Saratov Region	Wheat	2005
47	58242**	F. venenatum	Germany	Unknown	Unknown
48	58514**	F. venenatum	Leningrad Region	Oat	2013
49	58455**	F. venenatum	Novgorod Region	Wheat	2001
50	F-842***	F. sambucinum	Kiev region, Ukraine	Potato	1965
51	F-3966*** F. sambucinum^2^	Tula Region	Soil	2006	
52	F-4360***	F. sambucinum^2^	Buryatia	Wood	2005
53	64414**	F. equiseti	Kaliningrad Region	Barley	2006
54	65901**	F. equiseti	Leningrad Region	Barley	2006
55	97001**	F. equiseti	North Ossetia	Wheat	2007
56	F-3549***	F. equiseti4	Negev Desert, Israel	Soil	1995
57	F-2681***	F. incarnatum	Moscow Region	Unknown	1966
58	F-846***	F. sp^3^	Moldova	Melon	1958
59	F-892 (ATCC36015)****	F. graminearum	USA	Unknown	1977
60	F-900****	F. sambucinum^2^	Krasnoyarsk Region	Larix sibirica	Unknown

^*^ – strains from the collection of the All-Russian Research Institute of Phytopathology;

^**^ – strains from the collection of the All-Russian Institute of Plant Protection;

^***^ – strains from the All-Russian Collection of Microorganisms;

^****^ – strains from the All-Russian Collection of Industrial Microorganisms.

^1^ – initially identified as F. graminearum;

^2^ – initial identification was not confirmed;

^3^ – initially identified as F. poae.

**Table 2:** Primers used for the chemotyping of type B trichothecene-producing strains.

Primer set	Sequence	Product length, bp	Tm, °C	Chemotype	Reference
12-1	12CON (univ.): 5’-CATGAGCATGGTGATGTC- 3’				[48]
	12 NF: 5’-TCTCCTCGTTGTATCTGG-3’	840	60	NIV	
	12-15F: 5’-TACAGCGGTCGCAACTTC-3’	670	60	15-ADON	
	12-3F: 5’-CTTTGGCAAGCCCGTGCA-3’	410	60	3-ADON	
12-2	3ADONf: 5’-AACATGATCGGTGAGGTATCGA-3’ 3ADONr: 5’-CCATGGCGCTGGGAGTT-3’	60	60	3-ADON	[42]
	15ADONf: 5’-GTTTCGATATTCATTGGAAAGCTAC-3’ 15ADONr: 5’-CAAATAAGTATCGTCTGAAATTGGAAA-5’	57	60	15-ADON	
	NIVf: 5’-GCCCATATTCGCGACAATGT-5’ NIVr: 5’-GGCGAACTGATGAGTAACAAAACC-3’	77	60	NIV	
13-1	Tri13P1: 5’-CTCSACCGCATCGAAGASTCTC-3’ Tri13P2: 5’-GAASGTCGCARGACCTTGTTTC-3’	859 644 583	62	15-ADON 3-ADON NIV	[49]

un. – universal primer

T_m_ – melting temperature

In the case of system 12-2, the analysis was performed with each pair of primers separately, not in a multiplex PCR [48] format, to increase analysis specificity and avoid the formation of nonspecific amplicons.

The obtained results were confirmed by quantitative PCR (qPCR) with primer pairs from systems 12-1 and 12-2. In addition to standard components, 1.5 μL of 20× EvaGreen dye (Biotium, USA) was added to the reaction mixture. Amplification and fluorescent signal detection were performed in a DT-96-detecting amplifier (DNA-Technology, Russia). The PCR results were expressed as quantification cycles (C_q_, [50]). Each sample was analyzed in two independent replicates.

**HPLC analysis of toxin production by type B TrMT-producing strains**

To determine the type of TrMTs produced by the studied strains, fungal cultures were grown on a MYRO liquid medium [51] at 25 °C and 220 rpm for 5 days. The ability of isolates to produce DON and its monoacetylated derivatives was determined by reverse phase high pressure liquid chromatography of the culture filtrate supernatant separated from the mycelium by centrifugation [52, 53]. An 8 mL aliquot of the culture liquid supernatant was diluted with a acetonitrile : water (1 : 1) mixture to 10 mL and passed through a 0.22 μm Millipore membrane filter; 10 μL of the sample was introduced into the injector of a Waters 1525 Breeze HPLC system equipped with a Waters 2487 UV detector (Waters, USA). Separation was carried out on a Symmetry C18 (150 × 4.6 mm) column thermostated at 27°C. Mycotoxins were eluted with an acetonitrile : methanol : water (1 : 1 : 8 v/v/v – mobile phase) mixture at a flow rate of 0.5 mL/min and detected at 254 nm. Commercial DON, 3-AcDON, and 15-AcDON (Sigma-Aldrich, USA) were used as standards; as a control, we used the filtrate of the MYRO uninoculated medium that was incubated simultaneously with cultivation of submerged fungal cultures under the above conditions.

## RESULTS

**Phylogenetic properties of genes, analysis of their partial sequences using the BLAST algorithm, and microscopic analysis of the morphology of strains with controversial identification**

The main phylogenetic properties of the analyzed genes are given in *Table 3*. The DNA of all strains was amplified with a TEF50F–590R primer pair, which resulted in a single 452 to 483 bp amplification product containing two 80 to 100 and 236 to 254 bp introns. Except for the insertions and deletions omitted in the evaluation of the phylogenetic properties, the length of the analyzed sequences was 392 bp, including 129 (32.9%) variable nucleotides. The number of parsimony informative characters was 115 (29.3%), and the number of haplotypes was 17. An analysis of the *TEF1α *gene sequences using the BLAST algorithm confirmed the initial species identification for 54 strains. Of the six strains without confirmed identification, three were initially classified as *F. sambucinum*. The *TEF1α *sequences of strains F-3966 (No. 51, *Table 1*), NRRL 52726 relating to the *F. tricinctum *species complex, and NRRL 52727 (*F. avenaceum*) were shown to be 99.3% similar. Similarity for the strain F-4360 (No. 52) to the *F. acuminatum *strain, NRRL 52789 was 99.545%. The *TEF1α *gene sequence from the F-900 strain (No. 60) was 100% similar to a fragment of this gene from the *F. commune *strain NRRL 52764. The initial morphological species identification of strain F-3549 (No. 56) as *F. equiseti *was not confirmed: the BLAST analysis revealed 99% similarity to the sequence of strain NRRL 34033 from a relatively rare species, *F. brachygibbosum*. The strain 58212 (No. 32), initially identified as *F. graminearum*, had 100% similarity of the *TEF1α *sequence to that of CBS 123751–123745 strains typical of *F. ussurianum*. The most interesting result was obtained through a marker sequence analysis of the strain F-846 initially identified as *F. poae*. Comparison with the *TEF1α *sequences of typical strains deposited in databases did not reveal 100% similarity to any of them. The closest sequence was that from the *F. polyphialidicum *strain F-0016 (DQ295144, 97% similarity).

**Table 3:** Phylogenetic characteristics of analyzed sequences.

Locus	SL, bp	GC, %	VS, %	PIS, %	HT	H_d_	P_i_
TEF1α	392	53.2	32.9	29.3	17	0.933	0.08872
TRI5	379	48.5	37.2	36.1	13	0.907	0.13586
TRI14	650	49.1	36.7	34.5	23	0.96	0.13510
TEF1α+TRI5+TRI14	1421	50	35.9	33.5	28	0.976	0.1233

SL – sequence length

VS – variable sites

PIS – parsimony informative sites

HT –haplotypes

H_d_ – haplotype diversity

P_i_ – nucleotide diversity

TRI5F-R primer pair provided DNA amplification for all studied strains except for Nos. 51, 52, 56, and 60. An amplification product of the *TRI5 *gene was 431– 440 bp long and contained one intron of 52–61 bp in length. The length of the analyzed sequences was 379 bp, including 141 (37.2%) variable sites and 137 (36.1%) parsimony informative sites; the number of haplotypes was 13. The BLAST-based sequence analysis confirmed the accuracy of species identification for 54 strains. A DNA amplification product of the strain 58212 had 99% similarity with a *TRI5 *gene fragment from *F. asiaticum *(strains NRRL 26156 and 28720). It should be noted that none of the databases contained records of complete or partial structures of this gene in *F. ussurianum *strains, but given the close relationship between *F. asiaticum *and *F. ussurianum* [7], this result seems reliable. A DNA amplification product of the strain F-846 was 98% similar to a partial *TRI5 *gene sequence from the *F. langsethiae *strain KF2640 (JF966259).

PCR with the TRI14F-R primer pair revealed no DNA amplification products in samples Nos. 51, 52, 56, and 60 (as in the case of the TRI5F-R pair), as well as No. 58 (F-846). The DNA of the other strains was amplified with the formation of 698 to 705 bp products containing a single 50- to 59-bp intron. The analyzed sequence length (without insertions and deletions) was 650 bp, of which 239 bp (36.8%) were variable, and 224 bp (34.5%) were parsimony informative; the number of haplotypes was 23. The search for similar sequences in the GenBank and Fusarium MLST databases showed that the studied strains of *F. graminearum *constitute two groups: the first group comprising Nos. 30 and 58 showed 100% similarity with the *TRI14 *sequence of strain CBS 138562 (KU572434.1), while the second group (Nos. 27–29, 31) was completely identical to the sequence of strain CBS 138561 (KU572429.1), with the sequence similarity in these two groups being 97%.

We performed a microscopic analysis of the main morphological structures of strains whose initial identification was not confirmed by the marker sequence analysis. In strains F-3966 and F-4360 growing on CLA, elongated curved macroconidia with three to four septa typical of *F. avenaceum*, *F. tricinctum*, and *F. acuminatum *[54] were revealed. The strain F-900 also formed curved macroconidia with four septa and oval microconidia about 10 μm in size – features typical of the species *F. commune *[55]. In the strain F-846, thick-walled microconidia with four to five septa, as well as oval and clavate microconidia, were found. This result confirms the suggestion about erroneous initial identification of the strain as *F. poae, *because this species is characterized by spherical or spinulose microconidia and rarely forms macroconidia, usually with three septa [54].

**Analysis of phylogenetic tree topology**

**Fig. 1:**
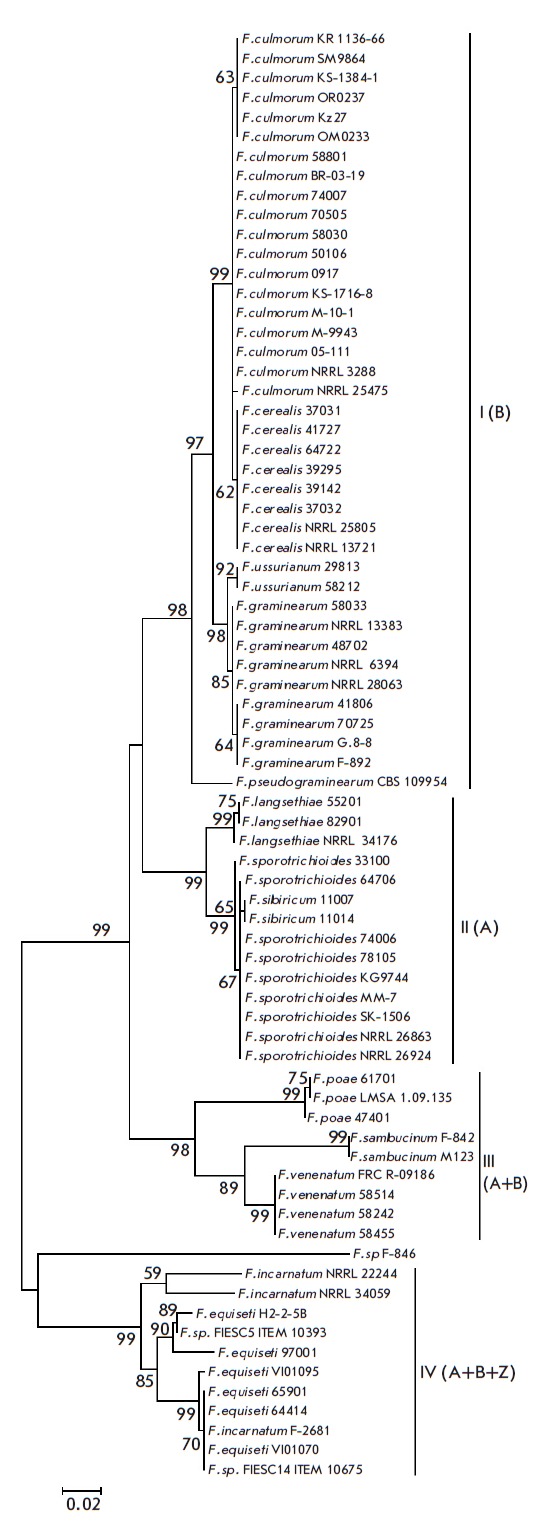
Phylogenetic tree constructed based on the alignment of partial *TEF1α *gene sequences of trichothecene-producing species using the maximum likelihood method (74 sequences). Only bootstrap values higher than 50% from 1,000 replicates are shown. 21 sequences from the NCBI GenBank are also included.

The phylogenetic trees generated on the basis of the structures of the three studied genes were characterized by both similarity and several significant topological differences. The *TEF1α *gene dendrogram (*Fig. 1*) comprises four large clusters supported by high bootstrap values (98 to 99%). Each cluster includes strains of species characterized by similar spectra of the produced mycotoxins. Cluster I (bootstrap support of 98%) is represented by type B TrMT-producing species (including *F. pseudograminearum *CBS109954, No. KM434220); cluster II (99%) is formed by type A TrMT producers; cluster III (98%) is represented by species producing both TrMT types; and cluster IV (99%) is represented by the species *F. equiseti *and *F. incarnatum *that are also capable of producing both type A and B toxins; however, unlike *F. poae *and closely related species, they also possess the ability to produce zearalenone and some other mycotoxins. It should be noted that clusters I–III and IV form separate groups supported by high bootstrap values (99%), and “*F. sp.*” (*Fusarium sp*. strain F-846) located in an intermediate position between these two groups. Clusters I and II include two subgroups: the first subgroup of cluster I (support 99%) comprises strains of the species *F. culmorum *and *F. cerealis*, and the second subgroup (98%) contains *F. graminearum *and *F. ussurianum*. Subgroups of cluster II are represented by the species *F. langsethiae *and *F. sporotrichioides*/*F. sibiricum *(bootstrap support of 99% each). Cluster IV is the most heterogeneous and represented by *F. equiseti *and *F. incarnatum *species. Two typical strains of *F. incarnatum *(NRRL 22244 and NRRL 34059) form a separate group supported by a bootstrap value of 59%, which, however, does not include the F-2681 strain investigated in this study. The *F. equiseti *strain 97001, together with strains H2-2-5B (JF496575) and 10393 (LN901566), forms a subgroup (90%), and strains 65901 and 64414 are included in another subgroup (99%) that also comprises strains VI01095 (AJ543560), VI01070 (AJ543562), and 10675 (LN901573).

**Fig. 2:**
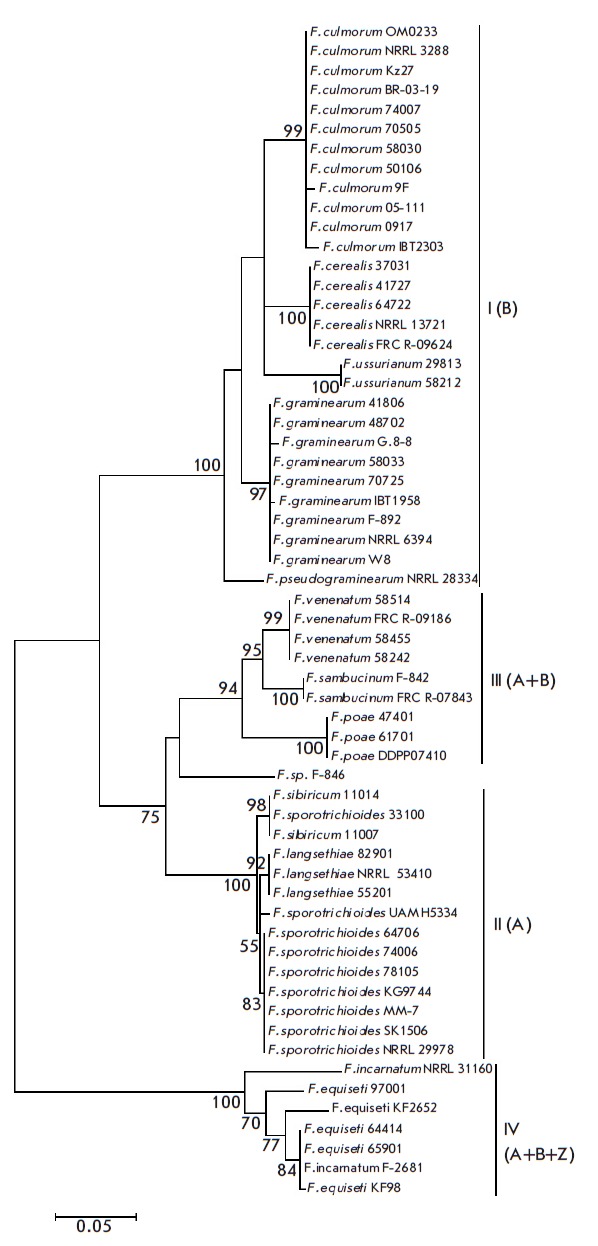
Phylogenetic tree constructed based on the alignment of partial *TRI5 *gene sequences of trichothecene-producing species using the maximum likelihood method (60 sequences). Only bootstrap values higher than 50% from 1,000 replicates are shown. 18 sequences from the NCBI GenBank are also included.

**Fig. 3:**
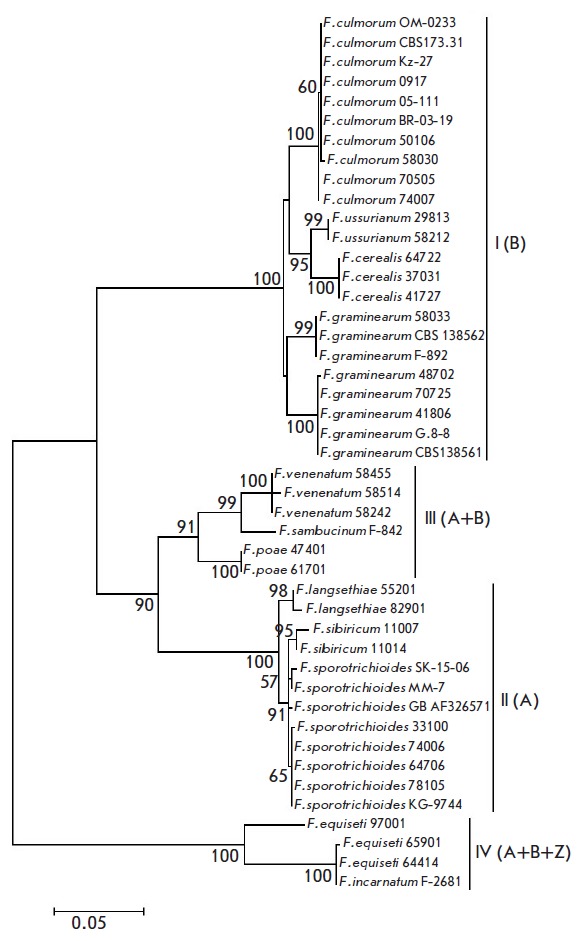
Phylogenetic tree constructed based on the alignment of partial *TRI14 *gene sequences of trichothecene-producing species using the maximum likelihood method (45 sequences). Only bootstrap values higher than 50% from 1,000 replicates are shown. 4 sequences from the NCBI GenBank are also included.

On the *TRI5 *gene dendrogram (*Fig. 2*), the bootstrap support of the main clusters corresponding to the toxigenic profiles of the studied species ranges from 94 (cluster III) to 100% (clusters I, II, and IV). In contrast to the *TEF1α *gene dendrogram, “*F. sp.*” is located in an intermediate position between clusters II and III. In addition, *F. sporotrichioides *strains do not form a single group but are distributed within cluster II; in particular, the strain 33100 belongs to the same subgroup as *F. sibiricum *strains (support of 98%). Cluster I lacks the *F. graminearum-ussurianum *subgroup characteristic of the *TEF1α *dendrogram. On the *TRI14 *gene phylogenetic tree (*Fig. 3*), *F. ussurianum *strains, together with *F. cerealis *strains, form a subgroup (bootstrap support of 95%), while *F. graminearum *strains are divided into two subgroups supported by bootstrap values of 99 and 100%, respectively. Cluster II includes the subgroups *F. langsethiae *(98%) and *F. sporotrichioides/ sibiricum *(91%); therefore, the topology of this cluster on the *TRI14 *dendrogram corresponds to the topology of *TEF1α *rather than the *TRI5 *dendrogram. It should be noted that in cluster IV, the *F. equiseti *strains 64414 and 65901 and *F. incarnatum *strain F-2681, on the one hand, and the *F. equiseti *strain 97001, on the other, form separate branches on the dendrograms of both *TRI *genes.

**Fig. 4:**
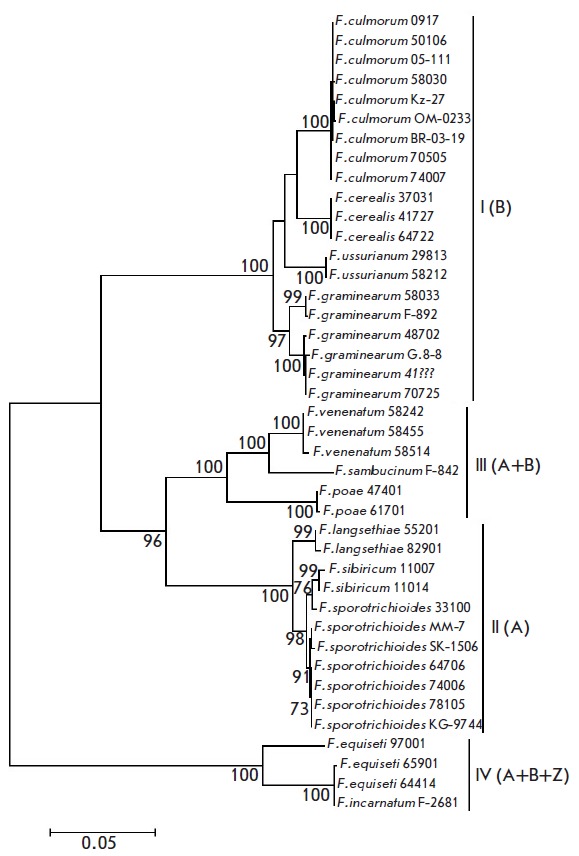
Phylogenetic tree constructed based on the alignment of combined (*TEF1α*+*TRI5*+*TRI14*) sequences of trichothecene-producing species using the maximum likelihood method (41 sequences). Only bootstrap values higher than 50% from 1,000 replicates are shown.

The structure of a phylogenetic tree generated based on the analysis of the combined sequence of the genes *TEF1α*, *TRI5*, and *TRI14* (*Fig. 4*) involves four large clusters supported by 100% bootstrap values. Of particular interest is a common group comprising clusters II and III (bootstrap support of 96%).

**Chemotyping of type B TrMT-producing strains and determination of mycotoxins by HPLC**

*Table 4* presents the results of the analysis of 33 type B TrMT-producing strains using qPCR with the primer sets 12-1 and 12-2. During the study, we decided to exclude set 13-1 from the use because an electrophoretic analysis of amplification products revealed two specific bands in all samples (data not shown): i.e., it was not possible to separate 3-ADON and 15-ADON chemotypes in *F. graminearum*. According to the chemotyping results, all analyzed *F. culmorum *and *F. ussurianum *strains belong to the 3-ADON chemotype, and the *F. graminearum *strains 58033 and 70725 also belong to it. The DNA of the *F. graminearum *strains G.8-8, 41806, and 48702 was amplified using a pair of 12CON– 12-15F primers, indicating that they belong to the 15- ADON chemotype (however, minor bands were also detected in samples of *F. graminearum *14-17, which was probably related to PCR conditions).

**Fig. 5:**
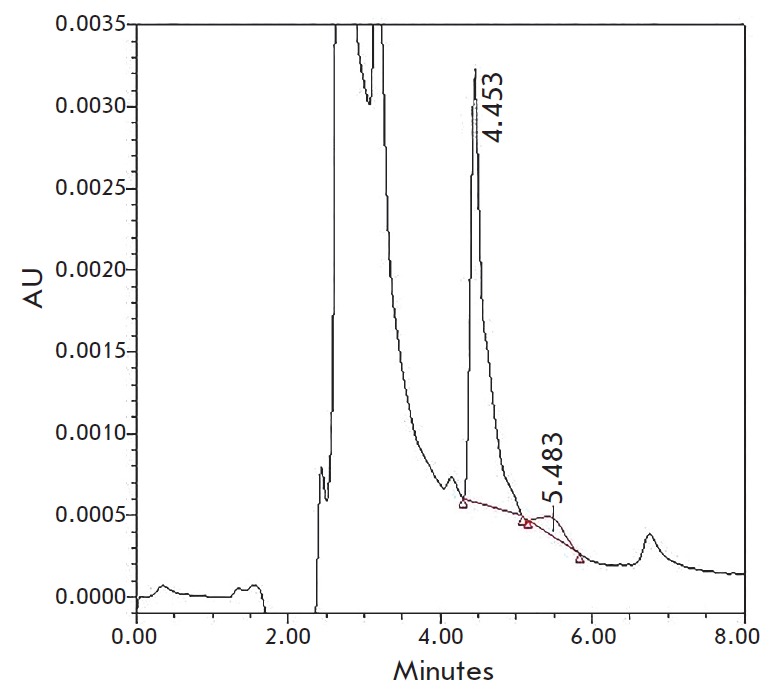
An HPLC chromatogram of a liquid culture medium of *F. ussurianum *29813. DON has a retention time of 4.453 min; 3-ADON has a retention time of 5.483 min.

An HPLC-based analysis of strains for the toxin-forming ability confirmed the molecular typing data for most samples (*Table 4*). In most cultures, DON quantitatively predominated over acetylated derivatives; no derivatives were detected in strains *F. graminearum *G.8-8, *F. culmorum *KP-1599-25/3 and KS-1384-1, and *F. ussurianum *58212. *Figure 5* shows an example chromatogram of the culture liquid of the *F. ussurianum *strain 29813, with peaks corresponding to DON (retention time, 4.453 min) and 3-ADON (retention time, 5.483 min).

## DISCUSSION

The main objective of this work was to study the genetic diversity and toxigenic characteristics of *Fusarium *fungal strains potentially capable of producing TrMTs, which were isolated in different regions of Russia and deposited in Russian national collections. Another important aspect of the study was to extend information about the structural features of the trichothecene cluster genes associated with the synthesis of the toxins and pathogenic properties of fungi.

Because the *TEF1α *gene is now considered to be the most studied and phylogenetically informative SCAR marker of genus *Fusarium *members, an analysis of its sequences has become the basis for verification of the taxonomic status of collection strains. The initial identification of six out of the 60 samples was not confirmed. The use of a combination of molecular genetic and morphological approaches enabled a highly reliable species identification for “controversial” strains. The strain 58212 (initially *F. graminearum*) was identified as *F. ussurianum*, and this fact correlates well with its geographic origin (Primorye region) and with the fact that species *F. graminearum *and *F. ussurianum *are almost indistinguishable by morphological features. Strains F-3966 and F-4360 deposited in collections as *F. sambucinum *were identified as *F. avenaceum *and *F. acuminatum*, respectively. Species *F. acuminatum *and *F. avenaceum *do not produce trichothecene mycotoxins; however, according to their ecological niches and growth pattern on potato sucrose agar, they are similar to *F. sambucinum*. There are no data on similar errors, but it is argued that *F. torulosum*, closely related to these two species, has been misidentified as *F. sambucinum *[54]. The strain F-900 isolated from forest tree nursery soil in the Krasnoyarsk region was identified as *F. commune *based on the results of a comprehensive study. The species *F. commune *described in 2003 [55] has not been detected to date in Russia. According to the available data, *F. commune *can be both a soil saprophyte and a pathogen of various plants, including economically important crops. *F. commune *is considered to be taxonomically close to the *F. oxysporum *species complex, but it is incapable of producing TrMTs [56], which is confirmed by the absence of PCR amplification products with primer pairs to the *TRI5 *and *TRI14 *genes. Based on an analysis of *TEF1α *and *TRI5 *gene sequences, the strain F-846 isolated from melon (Moldova, 1958), which was referred to as *F. poae *according to the Russian National Collection of Microorganisms, had no 100% similarity with any of the sequences deposited in databases. According to its marker gene structure, the strain was most close to the strains *F. polyphialidicum *F-0016 (*TEF1α*) and *F. langsethiae *F2640 (*TRI5*). The relationship between F-846 and *F. polyphialidicum *was not confirmed by an analysis of their morphological structures. Regarding the strain F2640 deposited in the GenBank, there are doubts about the accuracy of its identification as *F. langsethiae*, which are confirmed by significant differences in the *TRI5 *gene structure between this strain and typical strains of *F. langsethiae*. On dendrograms of the *TEF1α *and *TRI5 *genes, F-846 was located in an intermediate position and was not assigned to any of the large clusters. We may tentatively suggest that the strain F-846 is a separate phylogenetic species; however, confirmation of this suggestion requires additional analysis using a broader spectrum of DNA markers, investigation of its toxin-producing ability, and additional morphological and physiological data.

**Table 4:** Results of qPCR with primer sets 12-1 and 12-2 for 33 strains of trichothecene B-producing Fusarium species. «?» - minor bands on electrophoresis gel or minor amplification on last cycles are detected; «n/a» - not analyzed.

Strain	3-ADON			15-ADON			NIV			HPLC
Primer set	12-1	12-2	12-1	12-2	12-1	12-2	
F. culmorum M-99-43	+	+	-	-	-	-	3-ADON
F. culmorum 09-1/7	+	+	-	-	-	-	3-ADON
F. culmorum M-99-9	+	+	-	?	-	-	3-ADON
F. culmorum M-10-1	+	+	-	-	-	-	n/a
F. culmorum BR-03-19	+	+	-	-	-	-	n/a
F. culmorum BR-0453	+	+	-	-	-	-	n/a
F. culmorum OM-0233	+	+	-	-	-	-	n/a
F. culmorum OR-02-37	+	+	-	-	-	-	3-ADON
F. culmorum CM-9864	+	+	-	-	-	-	3-ADON
F. culmorum KP-1136-66	+	+	-	-	-	-	n/a
F. culmorum KP-1599-25/3	+	+	-	-	-	-	DON
F. culmorum KS-1384-1	+	+	-	-	-	-	DON
F. culmorum M-05-111	+	+	-	-	-	-	3-ADON
F. culmorum KC-1716-8	+	+	-	-	-	-	n/a
F. culmorum 58801	+	+	-	-	-	-	3-ADON
F. culmorum Kz-27	+	+	-	-	-	-	n/a
F. culmorum 74007	+	+	-	-	-	-	3-ADON
F. culmorum 58030	+	+	-	-	-	-	3-ADON
F. culmorum 70505	+	+	-	-	-	-	3-ADON
F. culmorum 50106	+	+	-	-	-	-	3-ADON
F. cerealis 64722	-	-	-	-	+	+	n/a
F. cerealis 39295	-	-	-	-	+	+	n/a
F. cerealis 37032	-	-	-	-	+	+	n/a
F. cerealis 39142	-	-	-	-	+	+	n/a
F. cerealis 37031	-	-	-	-	+	+	n/a
F. cerealis 41727	-	-	-	-	+	+	n/a
F. graminearum G.8-8	-	-	+	+	-	-	DON
F. graminearum 41806	?	?	+	+	-	-	15-ADON
F. graminearum 48702	-	?	+	+	-	-	15-ADON
F. graminearum 58033	+	+	-	-	-	-	3-ADON
F. graminearum 70725	+	+	-	-	-	-	3-ADON
F. ussurianum 58212	+	+	-	-	-	-	DON
F. ussurianum 29813	+	+	-	-	-	-	3-ADON

In recent years, the species *F. equiseti*, together with the closely related *F. incarnatum*, has been shown to have a complex phylogenetic structure, forming a heterogeneous group called the *F. incarnatum-equiseti *species complex (FIESC). *F. equiseti *strains from Northern and Southern Europe form two different groups named type I and II [57]. In the present study, marker sequences of the *TEF1α *gene of strains from different regions of Russia were compared with sequences of this gene from strains VI01070 and VI01095 belonging to type I, strain H2-2-5B belonging to type II, and strains 10675 and 10393 analyzed in [40]. Sequence comparison and phylogenetic analysis of *TEF1α *demonstrated that the strain 97001 appears to belong to type II (Southern Europe), which correlates with its geographic origin (North Ossetia), while strains 64414 and 65901 (Kaliningrad and Leningrad Region, respectively) belong to the North European type I. An exception to this rule was the strain 10675 (LN901573) isolated from wheat in Spain in 2009, assigned, however, according to the constructed tree topology, to type I. The *F. incarnatum *strain F-2681 also belonged to type I.

The use of structures of the genes responsible for various stages of mycotoxin biosynthesis in phylogenetic studies has been a controversial issue, because most of these genes have been acquired through horizontal transfer. Usually, a comparative analysis of these markers does not correctly reflect the evolutionary relationships among taxa, but it may be useful in identifying the features of toxin formation or the pathogenic properties of the strains. In this study, we investigated the partial structures of two trichothecene cluster genes: *TRI5 *encoding trichodiene synthase responsible for the first stage of TrMT biosynthesis, and *TRI14 *the exact function of which is unknown but is presumably associated with the regulation of DON biosynthesis or its transport beyond the cell during the development of the fungus in infected plant tissues [16]. The information on the sequences of the trichothecene cluster genes presented in databases is rather limited. For example, one *TRI5 *gene sequence from *F. venenatum *and *F. incarnatum *and two gene copies from *F. sambucinum *are deposited. There are only three *TRI14 *gene annotations for *F. sporotrichioides*, one annotation for *F. pseudograminearum*, and seven and four annotations among complete sequences of the trichothecene cluster of *F. graminearum *and *F. culmorum*, respectively. Therefore, for the first time, partial sequences of the *TRI14 *gene for 9 of 12 examined species were determined and deposited in the GenBank database. A characteristic feature of the *TRI5 *and *TRI14 *genes is the fact that a significant portion of the variable sites in the genes is located in the coding part, which is reflected in the differences in the amino acid sequences of appropriate proteins among and within species. The reverse is observed for the *TEF1α *gene, in which all variable sites occur in introns, and the coding part is highly conserved. We believe that these differences may reflect the different evolutionary history of these genes and also the fact that the translation elongation factor does not belong to proteins specific to the genus *Fusarium*, while the polymorphism of the amino acid sequences of the proteins encoded by the *TRI *genes has an important adaptive value. This may also explain the differences in topology of phylogenetic trees constructed based on *TEF1α*, *TRI5*, and *TRI14*. One of the differences is common branches formed by clusters II (type A TrMT producers) and III (TrMT A+B) on the dendrograms of the *TRI5 *and *TRI14 *genes, respectively, and supported by high bootstrap values (90 and 75%). In turn, on the *TEF1α *gene dendrogram, cluster II, along with cluster I (type B TrMT producers), forms a common branch, although the bootstrap support of this branch is low (less than 50%). Also, on dendrograms of the *TRI5 *and *TRI14 *genes, *F. ussurianum *strains form common clusters with strains of the species *F. culmorum *and *F. cerealis*; this contradicts the evolutionary data according to which *F. ussurianum *is most close to the species *F. graminearum *[22], which is confirmed by a phylogenetic analysis of *TEF1α*. On the *TRI14 *gene dendrogram, the studied strains of *F. graminearum *were divided into two clusters with high bootstrap support, but we could not refer this grouping either to the chemotype of the strains or to their geographical origin. We could assume a relationship between the *TRI14 *gene polymorphism and differences in the aggressiveness of different strains with respect to the host plant. However, similar studies were not performed in the present work. For the *TRI5 *gene, no correlation between *F. graminearum *chemotypes (3/15-ADON) and the species’ phylogenetic structure was found. In addition, none of the analyzed *F. graminearum *and *F. culmorum *strains contained an 8-nucleotide deletion (TGGAACAA), a marker for weakly toxigenic strains of these species [58].

Despite an approximately identical content of variable and phylogenetically informative sites, as well as haplotypic diversity of the three genes, an analysis of *TEF1α *more accurately reflected the phylogenetic structure and presumable evolutionary development of a group of *Fusarium *genus species TrMT producers. This result agrees with the data in an earlier study of the polymorphism of other genes involved in mycotoxin biosynthesis, e.g. *TRI1 *[7], or the gene of enniatin synthetase (*Esyn1*), a key enzyme of enniatin biosynthesis in *F. avenaceum *and closely related species [28, 59].

The belonging to a particular chemotype is a specific feature of a B-trichothecene producing strain. In recent years, studies have been published on the occurrence of chemotypes of B-trichothecene producers (primarily *F. graminearum*) in various regions of the world, e.g., in South America [60], Africa [61], and Europe [62, 63]. In 2016, the results of an extensive study conducted by experts from 17 European countries, including Russia, were published [64], which led to the creation of a European database (www.catalogueeu.luxmcc.lu). This database contains information on 187 strains isolated on the territory of Russia; however, it lacks information about strains from several regions, such as Western Siberia and the Volga-Vyatka Region, as well as information on the chemotypes of collection samples isolated in previous years. In the present study, the TrMT type was analyzed using a combined approach involving chemotyping with the chemotype-specific primers described earlier (see Molecular typing of type B TrMT producers) and an analysis of the culture fluids of some strains by HPLC. By using sets of primers (12-1 and 12-2) specific to the polymorphic regions of the *TRI12 *gene, we identified the chemotypes of the strains of four type BTrMT-producing species. It was demonstrated that the *F. culmorum *and *F. ussurianum *strains belong to the 3-ADON chemotypes, the *F. cerealis *strains belong to the NIV chemotype, and that there were both 3-ADON and 15-ADON producers among *F. graminearum *strains. It should be taken into account that PCR analysis data in some cases may not coincide with the actual toxigenic profile of a strain, which emphasizes the need to confirm genetic data by chemical methods [65]. HPLC confirmed the results of chemotyping for 16 out of 20 strains selected for chromatographic analysis. The culture liquids of four strains contained only DON, and acetylated derivatives were absent. The data published in recent years have demonstrated that 3-ADON producers predominate among *F. graminearum *strains of different geographical origins, which is mainly related to their higher aggressiveness compared to that of 15-ADON and NIV producers [66, 67]. An analysis of European strains of *F. graminearum *has demonstrated that the 3-ADON chemotype is common in Northern Europe, while the 15-ADON chemotype is more common in Central and Southern Europe [62-64]. Of the three studied *F. graminearum *strains with the 15-ADON chemotype, two may be assigned to the Central European group (G.8- 8 – Germany, 48702 – Tula Region), and one may be assigned to the South European group (41806 – North Ossetia). 3-ADON chemotype strains may be assigned to the North European (58033 – Leningrad Region) and Central European (70725 – Orel Region) groups. All the studied cultures of the species *F. culmorum *were assigned to the 3-ADON chemotype. It is believed that only two of the three known B chemotypes, 3-ADON and NIV, are characteristic of *F. culmorum*, with 3-ADON being much more ubiquitous [61, 64], which was confirmed in the present study. The *F. ussurianum *strain 29813 representing the 3-ADON chemotype produced the largest amount of DON among all the analyzed cultures. The relation of *F. cerealis *strains to the NIV chemotype is also in line with the data in [68]. We believe that the results obtained in the present study can partially compensate for information absent in the European database on the chemotypes of strains from certain regions of Russia. For example, strains from the Volga-Vyatka Region (No. 10–12), as well as Western Siberia and its neighboring Kostanay Region of Kazakhstan (No. 7, 16), were characterized for the first time.

## CONCLUSION

The findings of this study have both a basic and applied significance. The fundamental significance is associated with the great expansion of information on the molecular genetic diversity of trichothecene-producing strains of the genus *Fusarium*, which have different origins and represent different regions of Russia. The use of a complex approach combining the classical study of morphological structures with the analysis of highly informative DNA markers made it possible to verify and clarify species identification for a number of collection samples, in particular, to discover the first *F. commune* strain on the territory of Russia. For the first time, the *TRI14* gene was used for phylogenetic studies; the gene analysis revealed, on the one hand, a high level of interand intraspecific polymorphism and, on the other hand, the need for further investigations of its structure and functions, which would provide better understanding of its role in pathogenesis and mycotoxin biosynthesis. The applied significance of this study is related to the possibility of using the studied markers for developing monitoring systems for food contamination by TrMT producers. A combined study of the relation of type B TrMT producers to the main chemotypes using specific PCR and HPLC enabled an evaluation of their occurrence in Russia, including regions where TrMT producers had not been previously found.

## Abbreviations

PCR: polymerase chain reaction
TrMT: trichothecene mycotoxin
TEF1α: translation elongation factor 1-α gene
TRI5: trichodiene synthase gene
bp: base pairs
